# Role of Lnc-RNAs in the Pathogenesis and Development of Diabetic Retinopathy

**DOI:** 10.3390/ijms241813947

**Published:** 2023-09-11

**Authors:** Sofia Perisset, M. Constanza Potilinski, Juan E. Gallo

**Affiliations:** 1 Instituto de Investigaciones en Medicina Traslacional (IIMT), Facultad de Ciencias Biomédicas, Universidad Austral—CONICET, Pilar B1629, Buenos Aires, Argentina; sofiaperisset@gmail.com (S.P.); cpotilinski@austral.edu.ar (M.C.P.); 2 Departamento de Oftalmología, Hospital Universitario Austral, Pilar B1629, Buenos Aires, Argentina

**Keywords:** Lnc-RNAs, diabetic retinopathy, blindness, genomics, gene therapy, retina, precision medicine

## Abstract

Important advances in diabetic retinopathy (DR) research and management have occurred in the last few years. Neurodegenerative changes before the onset of microvascular alterations have been well established. So, new strategies are required for earlier and more effective treatment of DR, which still is the first cause of blindness in working age. We describe herein gene regulation through Lnc-RNAs as an interesting subject related to DR. Long non-coding RNAs (Lnc-RNAs) are non-protein-coding transcripts larger than 200 nucleotides. Lnc-RNAs regulate gene expression and protein formation at the epigenetic, transcriptional, and translational levels and can impact cell proliferation, apoptosis, immune response, and oxidative stress. These changes are known to take part in the mechanism of DR. Recent investigations pointed out that Lnc-RNAs might play a role in retinopathy development as Metastasis-Associated Lung Adenocarcinoma Transcript (Lnc-MALAT1), Maternally expressed gene 3 (Lnc-MEG3), myocardial-infarction-associated transcript (Lnc-MIAT), Lnc-RNA H19, Lnc-RNA HOTAIR, Lnc-RNA ANRIL B-Raf proto-oncogene (Lnc-RNA BANCR), small nucleolar RNA host gene 16 (Lnc-RNA SNHG16) and others. Several molecular pathways are impacted. Some of them play a role in DR pathophysiology, including the PI3K-Akt signaling axis, NAD-dependent deacetylase sirtuin-1 (Sirti1), p38 mitogen-activated protein kinase (P38/mapk), transforming growth factor beta signaling (TGF-β) and nuclear factor erythroid 2-related factor 2 (Nrf2). The way Lnc-RNAs affect diabetic retinopathy is a question of great relevance. Performing a more in-depth analysis seems to be crucial for researchers if they want to target Lnc-RNAs. New knowledge on gene regulation and biomarkers will enable investigators to develop more specialized therapies for diabetic retinopathy, particularly in the current growing context of precision medicine.

## 1. Introduction

Diabetic retinopathy (DR) is a neurovascular disease caused by hyperglycemia. Retinal degeneration initially included glial and neural cells [1,2]. The earliest clinical changes in response to the decrease in retinal perfusion in retinal blood vessels are dilatation and blood flow changes as a way of autoregulation to maintain retinal metabolism [3]. The disappearance of the capillary support cells (pericytes) through several mechanisms results in alterations in vascular regulation and stability that later lead to other abnormalities, such as vascular leakage and neovascularization (NV) [4]. In addition to pericyte loss, apoptosis of endothelial cells and thickening of the basement membrane are also detected during the progression of DR, contributing to blood–retina barrier (BRB) damage. This loss of vascular cells in the retina results in capillary occlusion and ischemia that in turn lead to transcription of proangiogenic factor VEGF through activation of hypoxia-inducible factor 1 (HIF-1) [3,5].

Although some advances in diabetic retinopathy management have occurred, the disease is still the first cause of blindness in working age [6,7]. Further efforts should be made to improve our knowledge of DR pathophysiology and to elaborate new strategic therapies.

## 2. Diabetic Retinopathy at a Molecular Level

### 2.1. Oxidative Stress

In hyperglycemia, reactive oxygen species (ROS) are overproduced in the retina and oxidative stress begins the disruption of the mitochondria, which leads to retinal capillary cell apoptosis [8]. In vitro studies have shown increased superoxide levels in hyperglycemic conditions and increased hydrogen peroxide content in retinal cells [9].

Oxidative stress can be both a cause and a consequence of the metabolic alterations that hyperglycemia brings about through the mechanisms mentioned above (the polyol and hexosamine pathways, the activation of PKC and the accumulation of AGEs) [8,9]. Besides these disorders, epigenetic modifications, alterations in the activity of transcription factors such as NF-κB and nuclear factor erythroid 2 related factor 2 (Nrf2) and hyperglycemia-mediated mitochondrial dysfunction are also related to the overproduction of ROS [8]. 

Nrf2 is a transcription factor in charge of regulating the expression of several antioxidant enzymes, and it plays a very important role in the protection of the retinal vasculature from oxidative stress. Furthermore, NRF2 is involved in mitochondrial homeostasis. However, a chronic state of hyperglycemia and oxidative stress overloads the NRF2 defense mechanism: the activity of NRF2-associated antioxidant enzymes was reduced in in vitro models (HO-1 in animal diabetic models and SOD, glutathione reductase (GR), glutathione peroxidase (GPx) and catalase in diabetic patients). This NRF2 deficiency leads to NOX2 activation and the production of ROS [8].

NRF2 also regulates the inflammatory response induced by diabetes by regulating the expression of NF-κB and COX2. A decrease in NRF2 levels leads first to a release of inflammatory cytokines because of the induction of NF-κB and then to vascular cell apoptosis thanks to the overexpression of proapoptotic factor Bax or TNF-α, both associated with NF-κB [8].

### 2.2. Neurodegeneration

The loss of vascular cells is well characterized in early DR; in particular, the presence of “pericyte ghosts” has been related to apoptotic changes. Pericyte death leads to the formation of acellular capillary microaneurysms, the first clinically evident lesion in the examination of a diabetic eye [10]. There are many pathways triggered by hyperglycemia that are known to harm these very important cells: the formation of AGEs, inflammatory cytokines, activation of PKC-δ, oxidative stress and Ang2-related pathways [4].

Caspases are enzymes of the group of cysteine proteases that are involved in inflammatory processes and apoptosis. Caspase-2, -8, -9 and -10 oversee the initiation of programmed cell death, and caspase-3, -6 and -7 are the “executive” caspases [11]. The intracellular programmed cell death process is mediated by the proapoptotic mitochondrial wall protein Bid (Bcl-2 protein family), in close communication with other molecules involved in programmed cell death, such as Bax and Bak molecules, which also belong to the Bcl-2 family. The presence of caspase-3, caspase-9, Bax, Bad and Fas was detected in retinal ganglion cells (RGCs) of diabetic patients, and the apoptotic changes observed in retinal ganglion cells are present from as early as one month after diabetes onset [11]. This means that neural apoptosis precedes vascular apoptosis and that, since neurons are unable to replicate, the continuous death of these cells may lead to neurodegeneration [11]. See Table 1.

The accumulation of ROS induced by hyperglycemia increases the pore permeability in the mitochondria and the release of cytochrome C and activates other proapoptotic factors to initiate cell death through the caspase family [12]. High protein expression of retinal NF-κB was detected at the beginning of diabetic retinopathy, and it remains active throughout the apoptotic process of retinal capillary cells [13,14].

Sirtuin 1 (SIRT1) is a deacetylase that plays a key role in the regulation of cell proliferation, apoptosis and inflammation, and it has an antiapoptotic effect by acting on P53. SIRT1 decreases mitochondrial damage by deacetylation of the protein P53, so it is unavailable to bind to BCL-1 family proteins like BAX, which would later translocate to the mitochondria and activate the caspase pathway in retinal pigment epithelial (RPE) cells. There are other factors inhibiting the process of apoptosis, such as protein kinase B (Akt), cyclooxygenase 2 (Cox-2) and myeloid leukemia cell differentiation protein (Mcl-1). Akt is a protein kinase involved in cell cycle modulation, and it interacts with a PI 3-kinase to promote cell survival. Furthermore, the activation of the PI 3-kinase/Akt pathway and the increase in the cytoplasmic levels of Cox-2 in RGC, RPE cells and the ciliary body epithelium were associated with higher cell survival rate. Cox-2 stimulates the synthesis of prostaglandins and the expression of Mcl-1 via the activation of the PI 3-kinase/Akt system [12].

### 2.3. Inflammatory Process

Diabetes causes increased local and systemic production of inflammatory molecules that lead to the activation of mechanisms such as the polyol pathway, increment in growth factors, accumulation of advanced glycation end products (AGEs) [15], activation of protein kinase C (PKC) and leukostasis. Consequently, microvascular damage, oxidative stress and superoxide overproduction occur, resulting in a vicious cycle of chronic inflammation [2]. Studies have shown that inflammatory cytokines like TNF-α, IL-6, IL-8 and IL-1β were significantly upregulated in diabetic patients, and their expression level correlated with the severity of DR [16]. Also, animal studies found that retinal leukostasis was decreased in diabetic mice deficient in TNF-α [17]. Furthermore, the expression of inflammatory proteins is regulated by gene transcription through the activation of proinflammatory nuclear transcription factors, such as Factor-kappa-B (NF-kB) [5]. Retinal capillaries of diabetic eye donors show increased numbers of retinal pericytes with activated NF-κB relative to non-diabetic donors [18]. There is plenty of evidence on the role played by microglia in the development of inflammation in diabetic retinopathy. Liou et al. [19] showed that the activation of the microglia starts very early, producing the excretion of proinflammatory cytokines and mediators of the inflammation chain. Langmann et al. [20] found that microglial cells promote an increase in retinal production of iNOS, IL-1β, macrophage inflammatory protein-**1** alpha (MIP-1α), IL-6 and macrophage colony-stimulating factor (M-CSF). Shelton et al. [21] showed an increase in IL-1β, IL-6, IL-8, IL-13, interferon gamma-induced protein 10 (IP-10), ICAM-1 and nitric oxide (NO) in Müller and endothelial cells, confirming their participation in the diabetic inflammatory process [22].

The complement system also has implications in diabetic retinopathy. Complement factor C5a receptor is expressed on Müller cells and is upregulated by hyperglycemia [23]. 

### 2.4. Angiogenesis

Angiogenesis is an extremely regulated process that balances proangiogenic (VEGF) and antiangiogenic factors (PEDF). The process involves endothelial cell migration and proliferation, vessel maturation and degradation of the extracellular matrix. When a proangiogenic environment is laid out, there is an abnormal growth of new fragile and leaky blood vessels. The most important cause of retinal NV is ischemia, but there are other factors, such as inflammatory mediators, that contribute by producing angiogenic cytokines and growth factors [5]. Vascular endothelial growth factor (VEGF) is a glycoprotein that functions as a vascular growth factor, regulating retinal vascular leakage and angiogenesis. Research results showed a significant correlation between VEGF in serum and vitreous samples of patients with DR and diabetic macular edema (DME). Also, VEGF and its receptor were found on epiretinal membranes extracted from the eyes of patients with diabetes, and a correlation between VEGF concentrations and retinopathy activity was established. Numerous studies analyzed serum levels of this protein and concluded that it may be a potential biomarker for controlling the development and progression of DR in a non-invasive manner. Tear sample research is also being conducted, with very promising results [24].

Pigment epithelium-derived factor (PEDF) is a glycoprotein with anti-inflammatory and antioxidant as well as antiangiogenic functions. Its antiangiogenic activities may be mediated directly by decreasing the expression of the *VEGF* gene and indirectly by reducing the availability of VEGF-receptor 1 (VEG-FR-1).

In diabetic retinopathy, when the BRB is altered, macromolecules leak from the intraretinal vasculature into the interstitial spaces of the retina, leading to macular edema. This increase in permeability is favored by hypoxia-related growth factors, inflammatory mediators and especially VEGF [4].

## 3. Gene Regulation as a New Approach 

### 3.1. Long Non-Coding RNAs 

Long non-coding RNAs (Lnc-RNAs) are non-protein-coding transcripts larger than 200 nucleotides. Lnc-RNAs regulate gene expression and protein formation at the epigenetic, transcriptional and translational levels and can impact cell proliferation, apoptosis, viability, immune response and oxidative stress [25]. The synthesis of Lnc-RNAs begins in the nucleus and depends on RNA Polymerase II and III. Lnc-RNA promotors can also overlap with epigenetic modifications that regulate transcription factor binding sites to moderate gene expression. 

Lnc-RNAs can be categorized into different groups based on genome location, morphology, sequence and function:

Genome location:

- Genic Lnc-RNAs: They are situated in exonic or intronic regions and are transcribed in distinct regions that pass across protein-coding sites; depending on their location, they can be defined as follows:
  ○ Sense Lnc-RNAs: They overlap one or more exons of neighboring mRNAs on the same protein-coding strand.
  Antisense Lnc-RNAs: They overlap one or more exons of neighboring mRNAs on the opposite non-coding strand.
  Intronic Lnc-RNAs: They originate only from the intronic regions of a protein-coding gene in either direction.
  Bidirectional Lnc-RNAs: They have promoters in common with protein-encoding genes but are transcribed in the opposite direction.
  Promoter upstream Lnc-RNAs: They are located upstream of a promoter.
  3’-UTR-associated Lnc-RNAs: They are transcribed from a protein-coding gene’s 3’-UTR region.
- Intergenic Lnc-RNAs: They are situated within the genomic interval between two genes [26], not intersecting with any protein-coding sites.

Also, cis-acting Lnc-RNAs act on genes located on the same chromosome, while trans-acting Lnc-RNAs affect genes on distant chromosomes 29 [27] and regulate gene expression through recruitment of proteins to target sites or sequestration of transcription factors away from targeted sites of transcription [28].

Because gene regulation by Lnc-RNAs occurs at many different levels through nuclear and cytoplasmic mechanisms, the localization of an Lnc-RNA may indicate its mode of action. Nuclear Lnc-RNAs are generally involved in direct transcriptional regulation, alternative splicing and organization of nuclear architecture by histone modification. On the other hand, cytoplasmic Lnc-RNAs regulate expression at the post-transcriptional level by “sponging” miRNA or interacting with RNA-binding proteins to determine the stability and translation potential of mRNAs [29]. However, there are some Lnc-RNAs that have been found in both cellular compartments, and recent reports are beginning to demonstrate that they may be present in the mitochondria as well [27].

Level of regulation of gene expression:

Lnc-RNAs can regulate gene expression at pre-transcriptional, transcriptional and post-transcriptional levels:

- Pre-transcriptional mechanisms involve the modification of genes without changing the DNA sequence of the organism. These modifications may be chromatin remodeling, genomic imprinting and X chromosome inactivation. Lnc-RNAs can regulate gene transcription via histone modification, including histone methylation, acetylation and ubiquitination. In addition, Lnc-RNAs directly bind to DNA methyltransferase (DNMT), which can lead to promoter methylation and can also affect the expression of genes by controlling chromatin looping and recruiting chromatin-modifying enzymes to the DNA [29].
- Transcriptional regulation occurs when Lnc-RNAs block the promoter region or interact with an RNA-binding protein to locate on the gene promoter region or regulate the activity of transcription factors [29].
- Post-transcriptional regulation involves the complementary pairing of Lnc-RNA with target mRNA sequences to control the rate of translation or lead to RNA degradation [29]. Lnc-RNAs can act as a precursor of some miRNAs to regulate gene expression, or they can act as endogenous competitive RNAs, binding to miRNAs and thereby upregulating the translation of the corresponding mRNAs [26].

Lnc-RNAs related to diabetic retinopathy are shown in Table 2.

Lnc-MALAT1: Metastasis-Associated Lung Adenocarcinoma Transcript 1 (MALAT1) is an Lnc-RNA mainly expressed in the nucleus and was originally described in relation to individuals at high risk for metastasis of non-small-cell lung tumors. It was later discovered that it is significantly upregulated in a wide range of tumors and that it promotes tumor cell proliferation, apoptosis, migration, invasion or the metastatic spread of tumor cells [30].

Several studies found that hyperglycemia causes a significant upregulation of Lnc-MALAT1 level in retinal endothelial cells and diabetic retinas and that silencing of MALAT1 decreases retinal vascularization, vascular leakage and retinal inflammation [30]. Furthermore, MALAT1 is upregulated in the vitreous humor, aqueous humor samples and fibrovascular membranes of diabetic patients.

Regarding the inflammatory process, studies have demonstrated that MALAT1 may be implicated in the upregulation of inflammatory molecules such as IL-6, TNF-α, IL-1β and MCP-1 by controlling the expression of histone methyltransferase polycomb repressive complex 2 (PRC2) that alters the chromatin state [31]. The selective targeting of MALAT1 via siRNAs resulted in a subsequent reduction in IL-6 and TNF-α mRNA and protein levels in human umbilical vein endothelial cells (HUVECs) [32]. 

Another study group showed that Amadori-glycated albumin (AGA) induced retinal microglial activation via a miR-124-dependent mechanism. Microglial activation is linked to leukostasis and the secretion of several proinflammatory molecules, including MCP-1. miR-124 may suppress AGA-induced MCP-1 expression by binding to the 3′-UTRs of MCP-1 mRNA in retinal microglial cells. This study group showed that MALAT1 knockdown inhibited MCP-1 release in the retinas of rats with DM and improved DR in vivo [33].

MALAT1 also plays an important role in maintaining the antioxidant defense system. Recent data show that hyperglycemia increases Sp1 binding at the MALAT1 promoter in retinal microvasculature, thus increasing Lnc-RNA MALAT1 expression. MALAT1, in turn, enhances the binding of Sp1 at the Keap1 promoter to activate its transcription. High levels of Keap1 impede Nrf2 nuclear movement, hindering the transcription of the antioxidant response enzymes. Inhibition of Lnc-RNA MALAT1 by its siRNA blocks Keap1 upregulation, freeing Nrf2 to translocate in the nucleus and help the transcription of antioxidant genes HO1 and Sod2. These results were obtained in vitro (human retinal endothelial Cells (HRECs)), in vivo (diabetic mice), and in retinal microvessels from human donors with diabetic retinopathy [34].

However, Lnc-RNA MALAT1 is mainly involved in diabetic retinopathy by regulating angiogenic pathways. Studies have shown that genetic ablation of MALAT1 in vitro inhibits the proliferation, migration and tube-formation ability of endothelial cells [35].

VE-cadherin is a cell adhesion molecule localized at the endothelial junction that plays an important role in angiogenesis and vascular permeability, and its expression was shown to be significantly increased in diabetic retinas. Moreover, high glucose could increase VE-cadherin expression in human retinal microvascular endothelial cells (hRMECs). miR-125b is a member of the miRNA family that is downregulated in DR and was recently reported to inhibit tube formation through translational suppression of VE-cadherin in endothelial cells. A study revealed that the expression of MALAT1 and VE-cadherin was triggered by HG while the expression of miR-125b was inhibited. Additionally, MALAT1 binding to miR-125b resulted in VE-cadherin activation. Knockdown of MALAT1 inhibited the proliferation, migration, tube formation and vascular permeability of hRMECs by restraining the VE-cadherin/β-catenin complex and decreasing the expression of neovascularization-related proteins VEGF, MMP2 and MMP9 [35].

Another group studied the MALAT1/miR-203a-3p axis in relation to angiogenesis. They found that the expression of MALAT1 was upregulated in the retinas of oxygen-induced retinopathy mice and hyperglycemia-stimulated hRMECs and that the expression of miR-203-3p was decreased. Their study demonstrated that miR-203a-3p overexpression decreased angiogenesis and reduced HIF-1α and VEGFA levels in hyperglycemia-stimulated HRMECs and showed that the decreased miR-203a-3p level could be reversed by MALAT1 silencing. The conclusion is that the MALAT1/miR-203a-3p axis may elevate HIF-1α and VEGFA levels and promote angiogenesis in DR by sponging miR-203a-3p [36].

Another molecule related to MALAT1 in diabetic retinopathy was glucose-regulated protein 78 (GRP78), in relation to endoplasmic reticulum stress (ERS). GRP78 is a major chaperone protein that facilitates protein folding and regulates ERS by its interaction with unfolded protein response stress sensors in the endoplasmic reticulum. Under diabetic conditions, ERS plays an important role in regulating cell metabolism, inflammation and survival. ERS induces an unfolded protein response (UPR) that is accompanied by upregulated levels of GRP78 and C/EBP homologous protein (CHOP), which are responsible for ERS-mediated apoptotic pathways. MALAT1 plays a role in regulating angiogenesis and inflammation by modulating ERS. A study revealed that MALAT1 and GRP78 expression in vascular endothelial cells (RVECs) may be elevated by hyperglycemia, and it showed that GRP78 overexpression led to angiogenesis, ERS and inflammation in RVECs. This study essentially showed that the downregulation of GRP78 by MALAT1 knockdown attenuated hyperglycemia (HG)-induced angiogenesis and inflammation in human RVECs [83].

MicroRNA-200b (miR-200b) is also being researched because of its relation to MALAT1 in DR. Several studies have shown that miR-200b is downregulated in diabetes and involved in angiogenesis and neovascularization. Han et al. presented an upregulated expression of YAP1, MALAT1 and VEGFA and downregulation of miR-200b-3p in diabetic mice. YAP1 is believed to play important functions in proliferation, migration and angiogenesis. The YAP1/MALAT1/miR-200b-3p/VEGFA axis was involved in the HG-induced angiogenesis of RMECs [83]. Their research provided evidence that YAP1 silencing alleviates DR development through the MALAT1/miR-200b-3p/VEGFA axis. Under the HG conditions, YAP1 silencing reduces the proliferation, migration and tube formation of retinal microvascular endothelial cells (RMECs); decreases the expression of MALAT1 and the VEGFA axis; and promotes the expression of miR-200b-3p [37].

Moreover, MALAT1 may regulate cell proliferation via p38 MAPK signaling pathways. Studies showed that MALAT1 knockdown significantly changes the levels of phosphorylated p38 MAPKs but has no effect on the levels of phosphorylated ERK1/2 or JNK1/2 and that its effects could be specifically blocked by a p38 MAPK pathway inhibitor or p38 siRNA. This indicates that there is a crosstalk between MALAT1 and p38 MAPK signaling, but further studies are needed to comprehend this association [30].

Lnc MEG3: Maternally expressed gene 3 (*MEG3*) is a non-coding transcript belonging to the imprinted DLK1-MEG3 locus located at chromosome 14q32. Its expression is decreased in several human tumors and tumor cell lines, and it affects a variety of biological processes, including chromosome assembly, epigenetics, protein translation and genomic defense. It has been shown that the MEG3 gene region on the chromosome increases susceptibility to type 1 diabetes and that epigenetic modifications of the DLK1-MEG3 miRNA cluster are also altered in human type 2 diabetic islets. 

In DR, MEG3 expression decreases in the serum of DR patients and HG-treated RPE cells, while MEG3 overexpression reduces VEGF and transforming growth factor-β1 (TGF-β1) expression. MEG3 knockdown also increases the proliferation, migration and tube formation of retinal endothelial cells in vitro [39].

Regarding the inflammatory process present in diabetic retinopathy, this Lnc-RNA has been extensively researched. A recent study in which an Lnc-RNA MEG3 vector was injected into the vitreous cavity of diabetic rats demonstrated that the expression levels of Fox01 and IL-1β can be reduced by this Lnc-RNA vector. Fox01 plays a major role in regulating oxidative stress, proliferation, apoptosis, differentiation and autophagy. Previous studies have shown that the binding between Fox01 and IL-1β can amplify the inflammatory response in DR by increasing the expression of the IL-1β receptor and binding to the IL-1β promoter, thus upregulating the expression of IL-1β in macrophages. Furthermore, it was demonstrated that Fox01 regulates the expression of IL-1β through Lnc-RNA MEG3. Zhao et al. determined that the expression of Fox01 was effectively inhibited and its binding to IL-1β was suppressed in DR rats transfected with Lnc-RNA MEG3, thereby alleviating DR. Immunohistochemical staining results revealed that the expression levels of Fox01 and IL-1β in the inner plexiform layer and inner nuclear layer were significantly upregulated in the DM group and Lnc-RNA MEG3 transfection group, but they significantly declined after Lnc-RNA MEG3 transfection. The results of Western blotting and qRT-PCR on the protein and mRNA expression levels of Fox01 and IL-1β in the retinas of diabetic rats showed the same results. Therefore, regulating this MEG3/Fox01/IL-1β pathway may be a potential new therapy for diabetic retinopathy [40].

A recent study revealed that after HG treatment of RPE cells, MEG3 promotes the expression of Sirt1 by acting as a sponge for miR-34a, thus inhibiting the activation of the NF-κB pathway triggered by HG and inhibiting the activation of Müller cells as well as the inflammatory response and apoptosis.

Another microRNA, miR-204, was identified as a Sirt1 upstream miRNA and an important regulator of Müller cells in DR. Research proved that inhibition of miR-204 by melatonin (MT) blocks the secretion of proinflammatory cytokines. So, it was concluded that MT inhibited the activation and secretion of proinflammatory cytokines in Müller cells and the progression of DR in vivo and in vitro, possibly through the MEG3/miR-204/Sirt1 pathway [39].

As stated above, SIRT1 could inhibit inflammation and apoptosis by regulating extracellular-signal-regulated kinase (ERK) and NF-κB pathways. Overexpression of SIRT1 could suppress the inflammation response, while its deletion leads to increased inflammation. Furthermore, the knockdown of miR-34a could alleviate the expression of inflammatory cytokines, and its expression was significantly upregulated in diabetes. In a diabetic environment, retinal epithelial cells expressed lower levels of MEG3 and SIRT1 and higher levels of miR-34a, meaning that diabetes could inhibit MEG3 and SIRT1 expression and increase miR-34a expression. However, studies have shown that overexpression of MEG3 decreased the miR-34a level and increased the SIRT1 level in ARPE-19 cells, indicating that MEG3 could negatively regulate the expression of miR-34a and positively regulate the expression of SIRT1. It was also demonstrated that overexpression of MEG3 and knockdown of miR-34a could inhibit the secretion of inflammatory cytokines such as IL-1β, IL-6 and TNF-α and the HG-activated NF-κB signaling pathway by blocking the expression of p-65 and inhibitor of NF-κB (IκB) in ARPE-19 cells. Furthermore, overexpression of MEG3 and knockdown of miR-34a determined an increased Bcl-2/Bax ratio, which resulted in decreased apoptosis. These results demonstrated that MEG3 could inhibit HG-induced inflammation and apoptosis signal pathways by inhibiting the NF-κB signaling pathway through downregulating miR34a [41].

Another pathway regulated by MEG3 is the miR-19b/SOCS6/JAK2/STAT3 axis. miR-19b is known to be involved in the regulation of cancer progression, and it was identified as a potential biomarker for diabetic cardiomyopathy. In research, miR-19b was discovered to be upregulated in HG-treated hRMECs, and this overexpression significantly inhibited cell viability and aggravated HG-induced apoptosis, stimulated inflammatory factors and suppressed the proliferation of hRMECs by targeting the SOCS6-mediated JAK2/STAT3 signaling pathway. Suppressor of cytokine signaling (SOCS) proteins have been proven to be intracellular inhibitors of the JAK/STAT pathway and regulate cytokine signaling by inhibiting JAK activity, and upregulation of SOCS3 inhibited MMP-9 expression in retinal microglia. miR-19b targeted and negatively regulated SOCS6 in DR. Xialo et al. confirmed that MEG3 suppressed high-glucose-induced cell apoptosis of hRMECs by negatively regulating miR-19b and that overexpression of MEG3 enhanced cell viability, reversing the inhibitory effect of miR-19b [42].

Another study revealed the anti-inflammatory and antiapoptotic functions of MEG3 through the miR-93/Nrf2 axis. There is an upregulated expression of miR-93 in DR patients, as well as in HG-exposed HRPE and ARPE-19 cells, indicating that miR-93 plays a potential role in the development of DR. This study also confirmed that miR-93 inhibited the viability and increased apoptosis and inflammation in HG-exposed ARPE-19 cells. *Nrf2* is a target gene of miR-93, and its overexpression alleviates apoptosis of ARPE-19 cells mediated by miR-93. Overexpression of MEG3 inhibited cleaved caspase-3 and Bax expression and upregulated the expression of Bcl-2, while overexpression of miR-93 reversed this trend. Likewise, knockdown of MEG3 stimulated the expression of IL-6 and TNF-α, whereas overexpression of miR-93 reversed the anti-inflammatory effect of *MEG3*. *MEG3* directly targets miR-93, and overexpression of *MEG3* alleviates HG-induced apoptosis and inflammation through the miR-93/Nrf2 axis [43].

Hyperglycemia results in a reduction in MEG3 levels in retinal endothelial cells and diabetic retinas that increases retinal angiogenesis and can aggravate vascular leakage and inflammation. MEG3 activates the PI3K/Akt signaling pathway and regulates retinal endothelial cell function in vivo and in vitro. The PI3K-Akt signaling axis is activated by hyperglycemia in endothelial cells and regulates multiple critical steps in angiogenesis, including endothelial cell survival, migration and capillary-like structure formation. Abnormal PI3K-Akt signaling could lead to abnormal cellular proliferation. Research shows that MEG3 knockdown has no effect on the amount of total Akt and PI3K, but it increases phosphorylated levels of Akt and PI3K. MEG3 knockdown promoted an increase in endothelial cell viability that was interrupted by PI3K inhibitors [44]. PI3K, AKT and mTOR were co-activated and involved in the regulation of cellular processes; PI3K could activate its downstream component AKT, which subsequently could induce the effector mTOR. 

DNA methyltransferase 1 (DNMT1) is a member of the DNA methyltransferase (DNMT) family. The aberrant expression of DNMTs could affect DNA methylation, which has been associated with the development of DR. Studies have shown that DNMT1 enhances the level of methylation in CpG islands of the MEG3 promoter and thus restrains the expression of MEG3 in renal fibrosis. This leads to activation of the PI3K/AKT/mTOR signaling pathway, thereby promoting endothelial–mesenchymal transition (EndMT) in DR, which results in the loss of expression of specific endothelial markers (VE-cadherin); acquisition of a mesenchymal phenotype; and production of mesenchymal cell products such as type I collagen, type III collagen and α-smooth muscle actin (α-SMA) [45]. EndMT has also been suggested to influence the development of fibroblasts and myofibroblasts, which are responsible for the progression of fibrosis and the development of proliferative DR [46].

Lastly, a TTR/PABPC1/MEG3/miR-223-3p signal axis was proposed to be related to the process of diabetic neovascularization. Transthyretin (TTR) is mainly expressed in human RPE cells and the choroid, and its serum and vitreous levels in diabetic patients were associated with DR progression. Also, TTR represses angiogenesis through the Tie signaling pathway and enhances the apoptosis of hRECs through a hypoxia-associated 78-kDa glucose-regulated protein (GRP78)-dependent pathway. In a study in which a vector containing TTR cDNA was injected into the vitreous of diabetic rats, the pathological progression of DR was partially reversed with overexpressed TTR, suggesting that TTR could repress the progression of DR. Also, direct binding of Lnc-RNA-MEG3 and miR-223 at two sites was proved in luciferase reporter assays, and overexpression or knockdown of MEG3 could significantly decrease or enhance the level of miR-223-3p, but miR-223-3p could not produce the same effects on Lnc-RNA-MEG3, which leads to the conclusion that miR-223 is a direct downstream target of Lnc-RNA-MEG3. Studies showed that miR223-3p was upregulated in the serum and aqueous humor of DR patients, and it was proved to suppress the proliferation of hRECs associated with FBXW7 and Notch1. In vitro, the addition of TTR could enhance the level of Lnc-RNA-MEG3, and overexpression of Lnc-RNA-MEG3 showed the same results for proliferation, migration, and tube formation properties of TTR in hRECs. Moreover, the knockdown and overexpression of PABPC1 could significantly decrease or promote the level of Lnc-RNA-MEG3, but Lnc-RNA-MEG3 showed no significant effects on PABPC, meaning that Lnc-RNA-MEG3 should be a downstream target of PABPC1. In conclusion, TTR could bind to PABPC1 and increase its content; PABPC1 could further bind and stabilize Lnc-RNA-MEG3, which could decrease the level of miR-223 and further promote the level of FBXW7, and then the Notch1 pathway could be inhibited [47].

Lnc MIAT: Lnc-RNA myocardial-infarction-associated transcript (MIAT), also known as retinal non-coding RNA 2 (RNCR2), has been associated with cell proliferation, apoptosis and migration in many diseases, such as myocardial infarction, microvascular dysfunction and diabetes. MIAT was confirmed to be implicated in the regulation of vascular function, including corneal angiogenesis, retinal angiogenesis and vascular leakage, and was also identified as a regulator of retinal neurodegeneration in diabetes [51]. Early retinal microvascular dysfunctions, such as blood flow disruption, basement membrane thickening, pericyte loss and acellular capillaries, were linked with upregulated MIAT expression and with increased plasma levels, while MIAT knockdown could partially reverse these pathological processes. A study showed that MIAT levels in endothelial cells of diabetic rats and in the fibrovascular membranes of diabetic patients were significantly higher than those detected in the non-diabetic controls [38]. Another study showed that in ARPE-19 cells, increased plasma levels of Lnc-RNA-MIAT only appeared in patients with diabetes combined with retinopathy but not in patients with diabetes without retinopathy, suggesting that Lnc-RNA-MIAT could distinguish between patients with and without diabetic retinopathy. Therefore, plasma levels of Lnc-RNA-MIAT may be a specific biomarker for diabetic retinopathy [52].

c-Myc is a protein that functions as a proto-oncogene and is overexpressed in many cancers. It has been reported that c-Myc could regulate a variety of biological activities in retinal Müller cells, such as proliferation, growth, and apoptosis. Researchers found that c-Myc was overexpressed both in DM rats and HG-induced Müller cells, thus implying that c-Myc might play important roles in the development of DR. Studies showed that upregulation of c-Myc contributed to the release of IL-1β, TNF-α and IL-6 from Müller cells by regulating the MIAT/TXNIP pathway, while c-Myc knockdown caused opposite results. c-Myc binds to the MIAT promoter and promotes MIAT expression, and MIAT promotes TXNIP by repressing its degradation. As an endogenous inhibitor of thioredoxin, TXNIP is implicated in the maturation of IL-1β and in inflammasome activation during DR, finally leading to oxidative stress, inflammation and apoptosis. This reveals that c-Myc promotes the release of proinflammatory factors from Müller cells by regulating TXNIP expression through MIAT [52].

Another study showed that in human retinal pigment epithelial cells cultured in an HG environment in vitro, increased expression of MIAT reduced cell viability through the activation of the TGF-β1 pathway. Increased levels of TGF-β have been discovered in the aqueous humor of diabetic patient eyes compared with normal eyes. However, while increased TGF-β signaling may be protective and may prevent the rapid progression of retinopathy, it also mediates inflammatory responses that promote the progression of diabetic retinopathy. Increased expression of MIAT significantly upregulated the expression of TGF-β1 in ARPE-19 cells and reduced cell viability, while treatment with a TGF-β inhibitor increased cell viability and reduced the inhibitory effects of Lnc-RNA-MIAT overexpression on cell survival. Also, a significant correlation between plasma TGF-β1 levels and Lnc-RNA-MIAT levels in patients with diabetic retinopathy has been described. However, TGF-β1 inhibition may not completely prevent endothelial cell damage associated with Lnc-RNA-MIAT overexpression, suggesting the possible involvement of other pathways in the Lnc-RNA-MIAT-mediated loss of cell viability [52].

Pyroptosis is a type of proinflammatory programmed cell death that depends on caspase-1 activation. A recent study showed that MIAT acts as a miR-342-3p sponge to regulate caspase-1 expression and consequent pericyte pyroptosis. miR-342-3p dysregulation has been observed in patients with type 1, type 2 and gestational diabetes mellitus and in diabetic nephropathy and vascular dysfunction. Moreover, advanced glycation end product–bovine serum albumin (AGE-BSA) treatment led to the upregulation of MIAT and CASP1, and MIAT knockdown notably reduced CASP1 and caspase-1 protein expression in pericytes. Research demonstrated that MIAT could compete with CASP1 for binding to miR-342-3p, thus alleviating the repressive effect on the expression of *CASP1*, the target gene of miR-342-3p. The results published indicate that MIAT functions as a sponge to regulate CASP1 expression by sequestering miR-342-3p, thereby promoting the caspase-1-dependent pyroptosis of human retinal pericytes [53].

Another apoptotic function associated with MIAT is through miR-29b and Sp1. miR-29b belongs to the miR-29 family involved in tumor suppression; Sp1 expression is directly targeted by miR-29b, which is bound to the miR-29b promoter and represses the expression of miR-29b. In turn, miR-29b inhibits the transcription of Sp1 and thereby upregulates its own transcription. Moreover, MIAT directly targeted miR-29b expression, and MIAT suppression significantly reversed the low expression of miR-29b and high expression of Sp1 induced by hyperglycemic conditions in Müller cells. MIAT suppression reversed apoptosis induced by high glucose, and miR-29b knockdown significantly reversed the positive effects of cell apoptosis induced by MIAT suppression. This leads to the conclusion that MIAT might function by partly absorbing miR-29b and inhibiting its function while regulating the expression of Sp1 [54].

Finally, MIAT has a regulatory effect on angiogenesis: here MIAT functions as a genome regulator at a transcriptional level, modulating VEGF levels by sponging miR-150-5p in retinal endothelial cells. During angiogenesis, MIAT is significantly upregulated, which alleviates the miR-150-5p repression effect, thereby upregulating the level of the miR-150-5p target gene, *VEGF*. Thus, Lnc-RNA-MIAT functions as a competing endogenous RNA (ceRNA), which may sequester miR-150-5p, thereby relieving its repressive effect on VEGF expression [55].

Lnc H19: The Lnc-RNA H19 derives from the paternally imprinted *H19* gene, and it is predominantly cytoplasmic, probably regulating gene expression at the post-transcriptional level. In diabetes, exposure to high glucose levels reduced H19 expression in neonatal cardiomyocytes and in the myocardium of diabetic rats. H19 expression was downregulated in retinal epithelial cells under high-glucose conditions and in vitreous humor samples from patients with PDR, and overexpression of H19 resulted in inhibitory effects on inflammation of ARPE-19 cells, suggesting that H19 suppressed the process of DR [58].

A recent study found that H19 might regulate the inflammatory processes of ARPE-19 cells under high-glucose conditions via enhancement of the expression of XBP1s through the inhibition of miR-93. X-box-binding protein 1 (XBP1) is a transcription factor activated by ERS and is involved in several cellular processes such as apoptosis and inflammation. ERS has been described to upregulate the expression of TNF-α and other inflammatory mediators during the development of diabetic retinopathy, and an elevated level of miR-93 in plasma has been associated with high risk of developing DR. This study found dysregulated expression levels of Lnc-RNA H19, XBP1s and miR-93 in the epithelial cells under high-glucose conditions and revealed that XBP1 might be the target of miR-93. So, it was concluded that Lnc-RNA H19 might upregulate the expression of XBP1 by sponging the XBP1-suppressor miR-93 in the ER [54].

Another inflammatory pathway associated with H19 is mediated by SIRT1 and miR-19b. Researchers found that overexpression of H19 inhibited the expression of TNF-α, IL-1β and IL-6 by binding to miR-19b to block HG-induced inflammatory responses in ARPE-19 cells. They also proved that miR-19b and SIRT1 mRNA could also bind together, and the expression of SIRT1 was controlled by miR-19b; so, H19 upregulated SIRT1 expression to suppress inflammatory responses by targeting miR-19b in ARPE-19 cells [48].

Lastly, studies showed that H19 overexpression prevented glucose-induced EndMT through TGF-β signaling via a blockade of the MAPK–ERK1/2 pathway; this action is independent of miR-200b, a miRNA previously shown to regulate diabetes-induced EndMT. TGF-β is known to be the major regulator of EndMT, and it induces its profibrotic properties by increasing fibroblast proliferation. It was also demonstrated that H19 regulated the production of phosphorylated ERK1/2 proteins, so H19 facilitates this regulation by suppressing TGF-β1 and its signaling pathways by repressing the MAPK–ERK1/2 signaling pathway [46].

Lnc HOTAIR: Lnc-RNA HOTAIR acts as an oncogenic molecule in different cancer cells, such as breast, gastric, colorectal and cervical cancer cells. It takes part in the epigenetic regulation of genes and plays an important role in different cellular pathways by interacting with PRC2 [59]. A study showed that HOTAIR expression was significantly increased in diabetic retinas and high-glucose (HG)-stimulated REC, and that is present in both nuclear and cytoplasmic compartments of ECs, making it possible for this Lnc-RNA to regulate the expression of several transcripts while simultaneously targeting enzymes involved in histone modification and DNA methylation. 

Another study showed that HOTAIR may be a potential therapeutic target for DR as it showed that knockdown inhibited the proliferation, invasion, migration, and permeability of HG-stimulated RECs in vitro and reduced retinal acellular capillaries and vascular leakage in vivo. It also contributes to glucose-induced mitochondrial and DNA damage and facilitates the epigenetic activation of VEGF-A by recruiting RNA polymerase II and acetylators such as P300 to the VEGF-A promoter. Also, HOTAIR can bind to lysine-specific histone demethylase 1(LSD1) and subsequently inhibit VE-cadherin transcription by reducing H3K4me3 levels on its promoter, which further facilitates HIF-1α-mediated transcriptional activation of VEGF-A [60], leading to VEGF-A overexpression and angiogenesis. Thus, HOTAIR may function as a molecular scaffold to regulate gene expression through interactions with epigenetic mediators [61].

Lnc ANRIL: Lnc-RNA ANRIL has been revealed to be a significant gene regulator in cardiovascular diseases, open-angle glaucoma, type 2 diabetes, intracranial aneurysm and several cancers. It has direct transcriptional effects or an indirect role as a recruiter of chromatin remodeling complexes like polycomb repressor complex 2 (PRC2) to specific genomic loci. PRC2 is one of the PRC transcription-repressive complexes (PRC1 and PRC2) that consist of many subunits (e.g., EZH2, EED, SUZ12 and RpAp46/48) and are critical in epigenetic regulation. For example, histone methylation involves the transfer of methyl groups to amino acid residues by histone methyltransferases such as EZH2. In some tumors, increased EZH2 promotes VEGF stimulation and subsequent angiogenesis. ANRIL has also been shown to regulate the histone acetylator p300 and to silence specific miRNAs.

It was determined that Lnc-RNA ANRIL is upregulated in response to high levels of glucose in diabetic retinopathy and the expression of Lnc-RNA ANRIL in the retinal tissues of DR rats was significantly elevated.

A recent study showed that ANRIL regulates glucose-mediated upregulation of VEGF through its interaction with p300 and PRC2 in glucose-cultured ECs and in the retinal tissue of diabetic animals. In ANRIL-silenced HRECs and in ANRIL knockdown mice, reduced levels of VEGF were accompanied by EZH2 reduction. Also, EZH2 blockade resulted in a reduction in ANRIL and VEGF RNA expression. ANRIL knockdown and HRECs following ANRIL siRNA transfection showed a reduction in p300 mRNA expression, and it had been previously revealed that there was increased production of p300 in diabetic retinopathy mediated by miR200b and that glucose-induced upregulation of VEGF was prevented by a miR200b blocker. The upregulation of miR200b in siANRIL-transfected HRECs suggests the involvement of ANRIL with mir200b in the regulation of VEGF. However, it is possible that additional molecules and mediators may be involved in these processes [62].

Another study showed that Lnc-RNA ANRIL regulates NF-κB in a rat model of DR. Knockdown of ANRIL was shown to significantly relieve damage to the retinas of rats and to decrease inflammation levels by reducing the expression of IL-1, IL-6 and MCP-1. Moreover, apoptosis in the retinal tissues of rats was suppressed significantly, and the microvascular function was improved. It was revealed that the expression and activation levels of P65 in the retinal tissues of DR rats declined prominently after Lnc-RNA ANRIL knockdown, suggesting that the inhibition of Lnc-RNA ANRIL can significantly ameliorate retinopathy in diabetic rats by inhibiting P65 in the NF-κB signaling pathway [63].

Lnc BANCR: Lnc-RNA B-Raf proto-oncogene, serine/threonine kinase-activated non-protein coding RNA (BANCR) is a well-studied Lnc-RNA in cancer biology, and it was recently proved to participate in retinoblastoma. Plasmatic levels of Lnc-RNA BANCR distinguished diabetic retinopathy patients from healthy controls, diabetic patients without obvious complications and diabetic patients with other complications. Also, it was demonstrated that, 12 months prior to the diagnosis of diabetic retinopathy, plasma levels of Lnc-RNA BANCR can differentiate patients who will develop diabetic retinopathy from healthy controls and diabetic patients without any obvious complications. Therefore, circulating Lnc-RNA BANCR may be a predictive biomarker for diabetic retinopathy [64]. In vitro experiments using ARPE-19 cells also suggested that Lnc-RNA BANCR expression was not affected by a high-glucose environment in the short term. BANCR overexpression inhibited the apoptosis of ARPE-19 cells under high-glucose treatment, and siRNA silencing reversed this process [65]. There is no research proving the expression of Lnc-RNA BANCR in aqueous or vitreous humor either in humans or animals, and the specific molecular mechanisms of BANCR in diabetic retinopathy remain unknown. 

Lnc SNHG16: Lnc-RNA small nucleolar RNA host gene 16 (*SNHG16*) has been studied in many cancers and was shown to promote angiogenesis of hemangioma endothelial cells [66]. A recent study on hRMEC revealed that SNHG16 was significantly upregulated after HG exposure in a dose-dependent and time-dependent pattern, suggesting a relation with DR. hRMEC proliferation was significantly stimulated by SNHG16 overexpression and inhibited by SNHG16 knockdown, and the levels of VEGF followed the same sequence: HIF-1α and VEGF levels were increased in hRMECs after SNHG16 overexpression but were reduced again by the knockdown of SNHG16.

A signaling pathway related to miR-146a-5p/IRAK1 and miR-7-5p/IRS1 activating NF-κB and PI3K/AKT was found to be responsible for proliferative DR-related abnormalities in cell proliferation, migration and angiogenesis in hRMECs. Because of the cytoplasmic location of SNHG16 in hRMECs, SNHG16 acted as a molecular sponge of both miR-146a-5p and miR-7-5p so they could, respectively, inhibit the NF-κB and PI3K/AKT pathways. This study found that both HG and SNHG16 upregulation increased the expression of IRAK1 and IRS1, activated NF-κB and PI3K/AKT and led to hRMEC dysfunction, so a molecular pathway related to SNHG16/miR-146a-5p/IRAK1 or SNHG16/miR-7-5p/IRS1 in DR was established [67].

Lnc HOTTIP: Lnc-RNA HOTTIP is a non-coding RNA transcribed from the HOXA family and was shown to be upregulated in retinal vascular cells and the retinas of diabetic animals in an HG environment. A recent study showed that HOTTIP promotes retinal inflammatory processes by activating p38-MAPK. HOTTIP silencing could significantly reduce retinal neovascularization and inflammation, and the knockdown of the HOTTIP gene inhibited proliferation and tube formation on vascular endothelial cells. This study revealed that the downregulation of HOTTIP can alter the expression level of phosphorylated p38-MAPK, and cell proliferation induced by HOTTIP can be inhibited by a p38-MAPK inhibitor or p38 siRNA [68].

Lnc NEAT1: Brain-derived neurotrophic factor (BDNF) is a nerve growth factor expressed in the brain and peripheral neural systems. It plays important roles in neural development and in the retina; it promotes cell differentiation, can inhibit inflammation, protects photoreceptors and ganglion neurons and prevents retinal neuronal degeneration under diabetic conditions. A recent study showed that BDNF was decreased in the retinas of DM rats and HG-treated Müller cells because it is negatively regulated by miR-497. In turn, miR-497 is a target of Lnc-RNA nuclear paraspeckle assembly transcript 1 (NEAT1). NEAT1 has been shown to be involved in many human diseases, including tumors and nervous system diseases, and it may have a regulatory role in type 2 diabetes. Under diabetic conditions, NEAT1 is downregulated, and the expression of BDNF is downregulated through elevating miR-497, leading to Müller cell apoptosis and aggravating DR [69].

However, another study found NEAT1 to be increased in hRECs under HG. Researchers reported that the knockdown of NEAT1 could inhibit DR progression via inactivating TGF-β1 and VEGF signaling [70]. So, further research must be conducted regarding this Lnc-RNA. 

Lnc BDNF-AS: The Lnc-RNA of BDNF-AS was recently discovered to be the natural antisense RNA of BDNF, and it may have common neural functions as BDNF. In the retina, BDNF-AS is abundantly expressed, and its inhibition was revealed to have neuroprotective effects against ischemic injury in retinal ganglion cells. A recent study showed that BDNF was significantly downregulated and BDNF-AS upregulated by 50 mM D-glucose in RPE cells. BDNF-AS knockdown prevented glucose-induced apoptosis and upregulated BDNF at both gene and protein levels, suggesting an antiapoptotic effect by BDNF-AS downregulation [71].

Lnc HEIH: Lnc-RNA HEIH was found to be highly expressed in the serum of patients with DR. This Lnc-RNA was previously known for its oncogenic role in hepatocellular carcinoma and colorectal cancer. HG induced ARPE-19 cell injury and increased the expression of HEIH. In turn, overexpression of HEIE aggravated cell injury and promoted apoptosis by releasing cytochrome C from mitochondria to the cytoplasm and enhancing the caspase-3 pathway. MicroRNA miR-939 might be a downstream target of HEIH, and VEGF was identified as a target of miR-939. VEGF activation triggers the PI3K/AKT signaling pathway, leading to the development and progression of DR. Research showed that the suppression of HEIH inhibited HG-induced activation of the PI3K/AKT signaling pathway, suggesting that HEIH may contribute to DR by sponging miR-939 to target VEGF expression and by regulating activation of the PI3K/AKT pathway [72].

Lnc IGF2-AS: Another natural antisense Lnc-RNA, insulin growth factor 2 antisense (IGF2-AS), was recently studied in relation to DR. It had been previously described in relation to several cancers, such as oral squamous cell carcinoma, hepatocellular cancer and lung cancer, and in hepatitis C, IGF2-AS was shown to regulate virus replication. A study revealed that IGF2-AS is significantly upregulated in ARPE-19 cells in an HG environment, in a concentration-dependent manner. Also, inhibition of IGF2-AS, by being transfected with IGF2-AS-specific siRNA, could protect glucose-induced apoptosis in RPE cells. Caspase-9, commonly upregulated by glucose in ARPE-19, was found to be reduced by IGF2-AS inhibition. Also, they found that IGF2 and AKT were significantly upregulated by this IGF2-AS inhibition in glucose-treated ARPE-19 cells, and AKT downregulation could reverse the protection of IGF2-AS inhibition on cell apoptosis. These results suggest that inhibition of IGF2-AS may act through the AKT signaling pathway to protect RPE cells against glucose-induced apoptosis [73].

Lnc SNHG7: Lnc-RNA small nucleolar host gene 7 (*SNHG7*) has been described as an important oncogene of various human cancers, including breast cancer, hepatic carcinoma and glioblastoma. In DR, this Lnc-RNA is downregulated under HG exposure, and it has repressive effects on cell proliferation, migration and angiogenesis. It also inhibits HG-induced EndMT. A recent study showed that Lnc-RNA SNHG7 blocked HG-induced hREC angiogenesis via a miR-543/SIRT1 pathway. Overexpression of SNHG7 significantly suppressed HRMEC proliferation, migration, and tube formation and VEGF expression in these cells. Upregulation of miR-543 or knockdown of SIRT1 could reverse the effects of SNHG7 in hRECs, suggesting that SHNG7 functions as an antiangiogenic gene via sponging miR-543 to positively regulate SIRT1 expression in hRECs [74]. Another pathway related to this Lnc-RNA is miR-34a-5p/XBP1. Research showed that XBP1 is a target of miR-34a-5p, and miR-34a-5p/XBP1 is a downstream signaling target of SNHG7, which results in protective effects on hRMECs. Loss of XBP1 leads to the activation of Müller glia cells and induces retinal inflammation in DR. XBP1 was found to be decreased in HG-treated hRMECs, and its knockdown promoted HG-induced EndMT and angiogenesis, suggesting an implication of the SNHG7/miR-34a-5p/XBP1 axis in DR [75].

Lnc SNHG4: Lnc-RNA small nucleolar RNA host gene 4 (*SNHG4*) was recently identified in osteosarcoma. It has been reported to regulate the production and release of multiple cytokines in inflammation. A recent study found that SNHG4 was downregulated in DR and may regulate HG-induced apoptosis by modulating the miR-200b/Oxr1 axis. However, SNHG4 is not downregulated in diabetic patients without obvious complications. SNHG4 could directly interact with miR-200b, and the interaction between Oxr1 and miR-200b is involved in the regulation of RPE cell apoptosis. The downregulation of Oxr1 after overexpression of miR-200b was also noted. So, this study group concluded that SNHG4 may sponge miR-200b to participate in DR [77].

Lnc RPSAP52: Lnc-RNA RPSAP52 is in the cytoplasm and was discovered to be related to pituitary tumors. Diabetic patients with DR have lower plasma levels of this Lnc-RNA compared with healthy controls, and in vitro studies in RPE cells also showed that glucose treatment downregulates RPSAP52. A recent study showed that overexpression of RPSAP52 reduced the apoptotic rate of RPE cells exposed to HG, suggesting a protective effect of this Lnc-RNA. The pathway suggested in this study includes Timp3, a protein that inhibits metalloproteinases, has positive effects on DR and is targeted by miR-365. It is proposed that RPSAP52 may sponge miR-365 to upregulate Timp3, decreasing RPE cell apoptosis [78].

Lnc KCNQ1OT1: Lnc-RNA KCNQ1OT1 expression was found higher in the aqueous humor of DR patients compared with normal controls, and miR-1470 was downregulated in DR patients and inversely correlated with the expression level of KCNQ1OT1. This study also showed that in hRECs, the level of miR-1470 was decreased and the level of KCNQ1OT1 was increased in HG conditions, and that KCNQ1OT1 promoted proliferation and angiogenesis in these cells, while knockdown of KCNQ1OT1 promoted apoptosis and inhibited cell proliferation. KCNQ1OT1 modulates EGFR by sponging miR-1470, and both the Akt pathway and EGFR are downregulated by KCNQ1OT1 knockdown and by miR-1470 overexpression [79].

Lnc FENDRR: FOXF1 adjacent non-coding developmental regulatory RNA (FENDRR) was found to be related to several forms of cancer such as breast, prostate and gastric cancer and is related to FOXF1 expression, a key factor of embryonic vasculature formation. Research found that FENDRR knockdown significantly reduced the expression of FOXF1, and FENDRR overexpression significantly increased the expression of FOXF1. In DR, FENDRR was increased in blood from DR patients and in high-glucose-exposed HRECs. In these cells, FENDRR promotes proliferation, migration, capillary formation and VEGF expression [80].

Lnc TDRG1: Human testis-specific gene testis development-related gene 1 (*TDRG1*) is an Lnc-RNA of interest due to its proliferative functions studied in bone marrow and endometrial carcinoma and its connection with VEGF-a. Regarding diabetes, it was found that this Lnc-RNA is overexpressed in fibrovascular membranes of patients with PDR when compared with epiretinal membranes of patients without diabetes. VEGF was also highly expressed in fibrovascular membranes when compared to controls, and in vitro studies in HRECs exposed to HG confirmed these results. Furthermore, the knockdown of Lnc-RNA TDRG1 in vitro reverted endothelial cell dysfunction caused by hyperglycemia, and a positive result in terms of cell proliferation and the integrity of tube formation of HRECs was proved. So, it is suggested that Lnc-RNA TDRG1 could protect endothelial cell proliferation during PDR by modulating VEGF [81].

Lnc UCA1: Long non-coding RNA urothelial carcinoma-associated 1 (UCA1) was found to be highly expressed in various cancers, stimulating the proliferation, migration and epithelial–mesenchymal transformation of tumor cells. Specifically in DM, it is upregulated in endothelial cells and was found to be implicated in diabetic nephropathy in rats. It is also upregulated in fibrovascular membranes and in the blood of patients with DR compared to those without retinopathy. In vitro studies in HRECs exposed to HG also follow these trends. It was also shown that the expression levels of miR-624-3p were reduced, leading to cell proliferation, migration and angiogenesis by promoting VEGF-C expression in endothelial cells. In conclusion, UCA1 may act as a sponge of miR-624-3p to stimulate high-glucose-induced neovascularization [82].

### 3.2. Main Involved Pathways 

**Figure 1:**
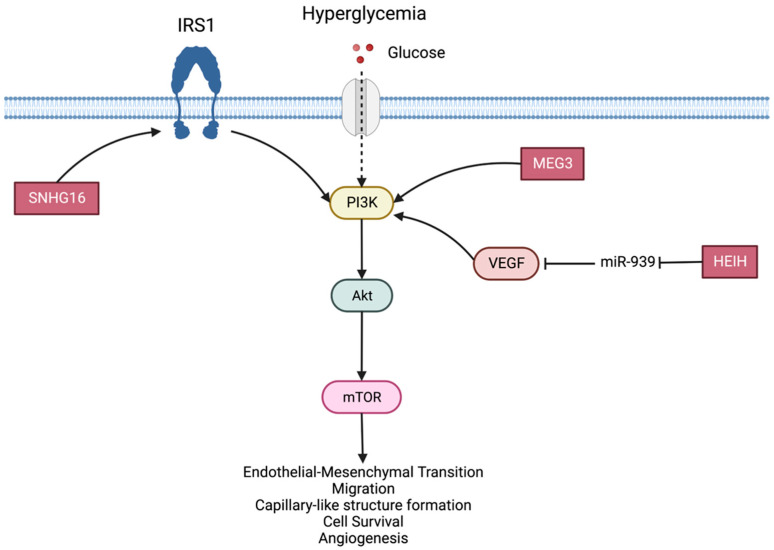
The PI3K/Akt signaling pathway. Role of Lnc-RNAs MEG3, SNHG16 and HEIH.

PI3k akt: The PI3K-Akt signaling axis is activated by hyperglycemia in endothelial cells and regulates multiple critical steps in angiogenesis, including endothelial cell survival, migration and capillary-like structure formation [44]. MEG3 activates the PI3K/Akt/mTOR signaling pathway and regulates retinal endothelial cell function, promoting endothelial–mesenchymal transition (EndMT) in DR (Figure 1) [46].

SNHG16 upregulation increases the expression of IRS1, activates PI3K/AKT and leads to hRMEC dysfunction. VEGF activation triggers the PI3K/AKT signaling pathway leading to the development and progression of DR. The suppression of HEIH inhibited HG-induced activation of the PI3K/AKT signaling pathway, suggesting that HEIH may contribute to DR by sponging miR-939 to target VEGF expression, thus regulating activation of the PI3K/AKT pathway in RPE cells [72].

SIRT1: After HG treatment of RPE cells, MEG3 promotes the expression of Sirt1 by acting as a sponge for miR-34a, thus inhibiting the activation of the NF-κB pathway triggered by HG and inhibiting the activation of Müller cells as well as the inflammatory response and apoptosis [41].

Researchers found that overexpression of H19 inhibited the expression of TNF-α, IL-1β and IL-6 by binding to miR-19b to upregulate SIRT1 in ARPE-19 cells [48].

**Figure 2:**
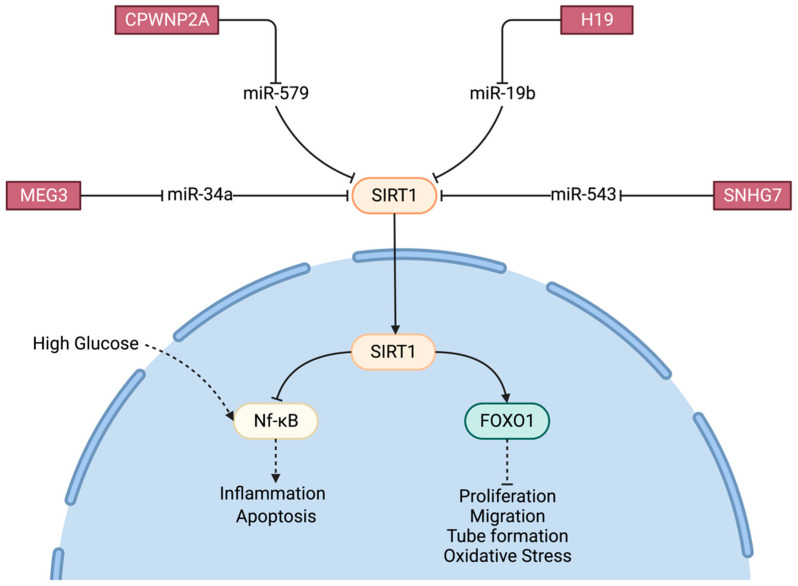
The SIRTI 1 pathway. Role of Lnc-RNAs MEG3, CPWNP2A, H19 and SNHG7.

Overexpression of SNHG7 significantly suppressed HRMEC proliferation, migration, tube formation and VEGF expression in these cells by sponging miR-543 to positively regulate SIRT1 expression in hRECs [74]. cPWWP2A acts as an endogenous miR-579 sponge to inhibit its function of decreasing the expression of angiopoietin 1, occludin and SIRT1 of pericytes of mouse retinas. This suggests that the cPWWP2A–miR-579–angiopoietin 1/occludin/SIRT1 network regulates retinal vascular dysfunction in vivo (Figure 2) [84].

**Figure 3:**
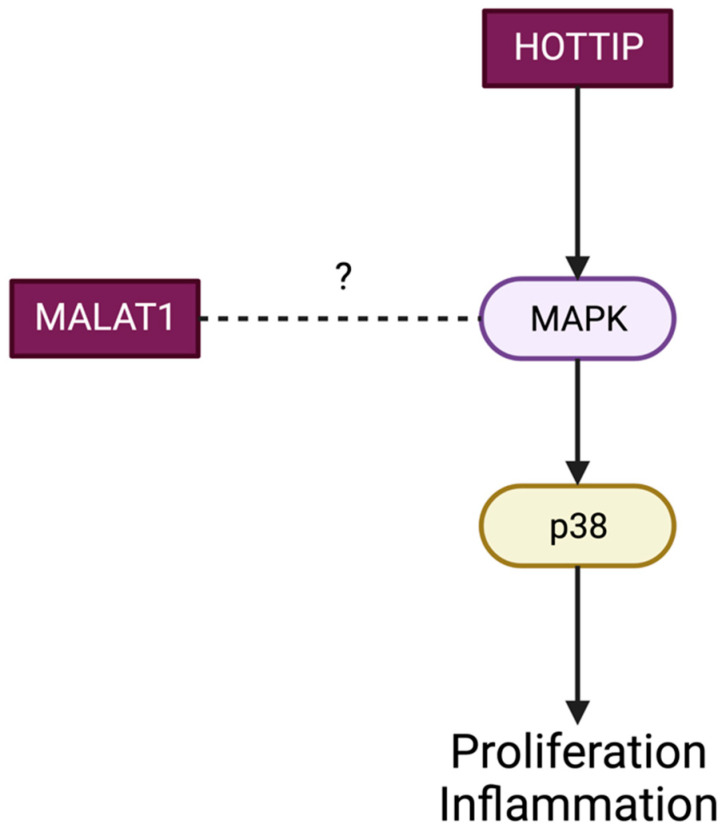
The P38/mapk pathway. A Mitogen-activated protein (MAP) kinase pathway plays an important role in regulating many cellular processes including inflammation, cell differentiation, cell growth and death. Role of Lnc-RNAs MALAT1 and HOTTIP.

P38/mapk: MALAT1 knockdown significantly changes the levels of phosphorylated p38 in chorioretinal cells, indicating that there is a crosstalk between MALAT1 and p38 MAPK signaling, but further studies are needed to comprehend this association (Figure 3) [30].

HOTTIP promotes retinal inflammatory processes and inhibits proliferation and tube formation on vascular endothelial cells by activating p38-MAPK [68]. H19 overexpression prevented glucose-induced EndMT by suppressing TGF-β1 and its signaling pathways by repressing the MAPK–ERK1/2 signaling pathway.

**Figure 4:**
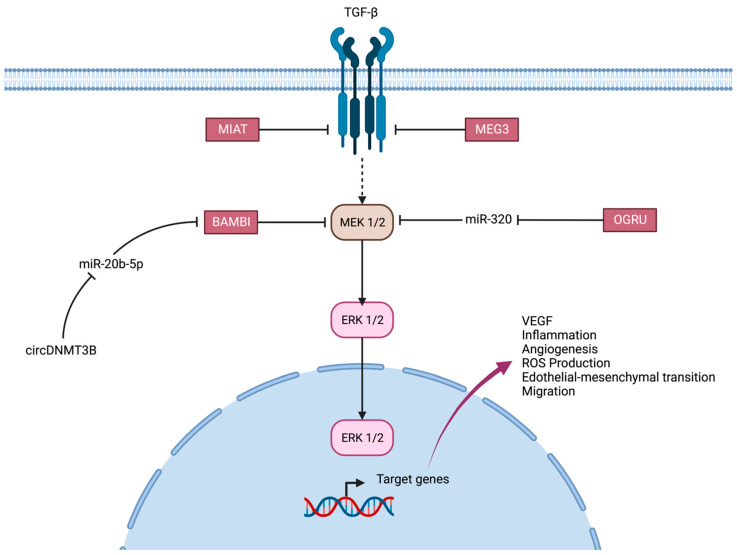
The TGF-β pathway. Role of Lnc-RNAs MIAT, MEG3, BAMBI and OGRU.

TGF-β: Increased levels of TGF-β have been discovered in the aqueous humor of the eyes of patients with diabetes compared with normal eyes [85]. However, while increased TGF-β signaling may be protective and may prevent the rapid progression of retinopathy, it also mediates inflammatory responses that promote the progression of diabetic retinopathy by disrupting angiogenesis and blood–retina barrier breakdown (Figure 4). TGF-β1 can also induce the expression of platelet-derived growth factors, fibroblast growth factors and VEGF to promote DR [49].

H19 overexpression prevented glucose-induced EndMT through TGF-β signaling via a blockade of the MAPK–ERK1/2 pathway. 

BAMBI, a type 1 TGFβ receptor antagonist, is downregulated in retinal endothelial cells under diabetic conditions and is regulated by miR-20b-5p and circDNMT3B: the decrease in BAMBI expression was reversed with a miR-20b-5p inhibitor, and overexpression of circDNMT3B alleviated the reduced expression of BAMBI under HG conditions [56].

In DR, MEG3 expression decreases in the serum of DR patients and HG-treated RPE cells, while MEG3 overexpression reduces VEGF and transforming growth factor-β1 (TGF-β1) expression. MEG3 knockdown also increases the proliferation, migration and tube formation of retinal endothelial cells in vitro [39].

In ARPE-19 cells cultured in an HG environment in vitro, increased expression of MIAT significantly upregulated the expression of TGF-β1 and reduced cell viability, but TGF-β1 inhibition did not completely prevent endothelial cell damage associated with Lnc-RNA-MIAT overexpression. Also, a significant correlation between plasma TGF-β1 levels and Lnc-RNA-MIAT levels in patients with diabetic retinopathy has been described [38].

Suppressing OGRU could improve miR-320 expression, exerting protective effects against HG-induced aberrant expression of VEGF and TGF-β1, inflammatory response and ROS production in Müller cells [50].

**Figure 5:**
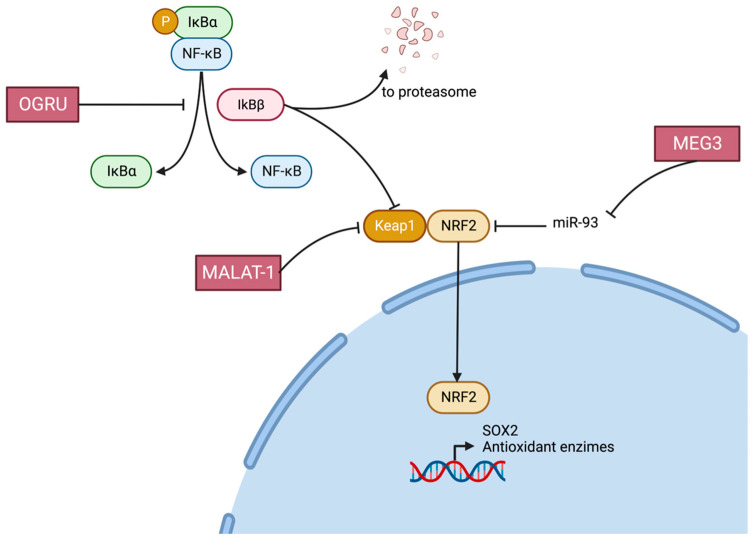
The nuclear factor erythroid 2 related factor 2 (NRF2) pathway. Role of Lnc-RNAs MALAT-1, MEG3 and OGRU.

**Figure 6:**
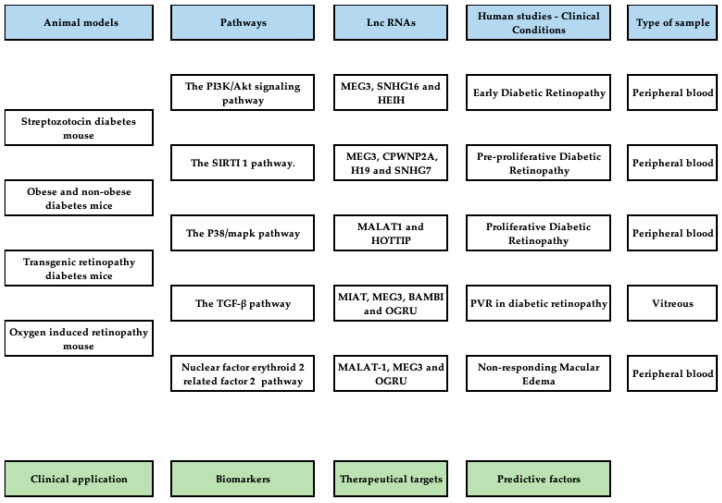
Diagram for future investigations on Lnc-RNA and diabetic retinopathy. Specific animal models should be used to further analyze Lnc-RNAs in five relevant pathways of DR development. Also, molecular and genetic evaluation of Lnc-RNAs in samples of humans, under specific clinical conditions, is recommended.

Nrf2: Nuclear factor erythroid 2 related factor 2 (NRF2) is a transcription factor in charge of regulating the expression of several antioxidant enzymes, and it plays a very important role in retinal vasculature protection from oxidative stress (Figure 5 and Figure 6).

MALAT1 activates Keap1′s transcription, thus impeding Nrf2 nuclear movement and inhibiting the transcription of the antioxidant response enzymes in HRECs, in vivo and in retinal microvessels from human donors with diabetic retinopathy [34].

*MEG3* directly targets miR-93, and overexpression of *MEG3* alleviates HG-induced apoptosis and inflammation through the miR-93/Nrf2 axis in RPE cells [43]. 

HG-induced inflammatory response and oxidative stress in vitro were markedly mitigated by OGRU knockdown through restraining IκBɑ/nuclear factor kappa beta (NF-κB) and improving nuclear factor erythroid 2-related factor 2 (Nrf2) signaling pathways, respectively, in Müller cells. 

Sox2OT knockdown plays a neuroprotective role in diabetes-related retinal neurodegeneration by regulating the oxidative stress response in RGCs and diabetic mouse retinas. Sox2OT knockdown plays an antioxidative role via regulating NRF2/HO-1 signaling activity.

JAK/STAT3: MEG3 ameliorated cell viability and decreased HG-induced apoptosis and inflammatory factors in hRMECs by negatively regulating miR-19b and thus targeting the SOCS6-mediated JAK2/STAT3 signaling pathway [42].

Circ_0005015 overexpression sponges miR-519d-3p, thus releasing the repressive effect of miR-519d-3p on STAT3, MMP-2 and XIAP in endothelial cells, which play important roles in many cellular processes such as cell growth, vascularization and apoptosis [57].

TPTEP1 is downregulated in HG-stimulated HRVECs, and its overexpression reduced viability, migration and angiogenesis. Moreover, TPTEP1 suppressed phosphorylation and nuclear translocation of STAT3 and thereby downregulated VEGFA mRNA and protein levels [86].

TXNIP: TXNIP is implicated in the maturation of IL-1β and in the inflammasome activation during DR, finally leading to oxidative stress, inflammation and apoptosis. c-Myc promotes MIAT expression, and MIAT represses TXNIP’s degradation, which leads to the release of proinflammatory factors from Müller cells [52].

circ_0084043 upregulated TXNIP expression to activate the Wnt/β-catenin signal pathway by targeting miR-128-3p, thus promoting HG-induced RPE cell injury [76].

XBP1: XBP1 is a transcription factor that is activated by ERS and is involved in several cellular processes such as apoptosis and inflammation. XBP1 was found to be decreased in HG-treated hRMECs, and its knockdown promoted HG-induced EndMT and angiogenesis. H19 might regulate the inflammatory processes of ARPE-19 cells under high-glucose conditions via upregulating the expression of XBP1 by sponging XBP1-suppressor miR-93 during ERS [58]. XBP1 is a target of SNHG7/miR-34a-5p/XBP1, with protective effects on hRMECs. Loss of XBP1 leads to the activation of Müller glia cells and induces retinal inflammation in DR [75].

## 4. Discussion and Conclusions

In this review, we have pointed out how Lnc-RNAs can be involved in diabetic retinopathy development. Despite efforts made by researchers, a lot of work still needs to be conducted to understand Lnc-RNAs’ influence on diabetic retinopathy. How Lnc-RNAs affect complex pathophysiological processes in the retina such as the onset and advance of diabetic retinopathy are questions of great relevance. In other medical diseases, such as asthma, Lnc-RNAs were discovered to be important post-transcriptional regulators [87]. In addition, a study underlined how MEG3 regulates Treg/Th17 balance in asthmatic patients [88]. Investigators expect to find Lnc-RNA biomarkers that distinguish asthma subtypes in a more clear and objective manner and to find new therapeutic targets to treat people with therapy-resistant asthma [58,75].

After ischemic stroke, Lnc-RNAs have been reported to stimulate apoptosis, angiogenesis and inflammation [89,90,91,92,93,94,95]. The brain responds by changing Lnc-RNA transcriptomic profiles. These Lnc-RNA abnormalities suggest the potential functional roles and predictive value of Lnc-RNAs as new biomarkers for stroke. A recent study found that the overexpression of MEG3 suppresses functional recovery after ischemia; the silencing of MEG3 ameliorates brain lesions; and the expression of MEG3 increases angiogenesis after ischemia by promoting endothelial cell migration, proliferation, sprouting and tube formation [94]. 

Targeting MALAT1 is a novel treatment strategy against cancer. Literature data on cancer indicate aberrant expression and dysregulated MALAT1 in several oncological diseases (citation). MALAT1 is an adverse prognostic marker in stage I lung adenocarcinoma and squamous cell carcinoma patients, and its expression is also associated with metastasis [96,97]. MALAT1 overexpression in breast cancer has been reported by different research groups [98,99]. MALAT1 was found upregulated in cervical cancer (CC) as compared to the normal cervix, and its expression correlated with worse overall survival in CC patients and associated with lymph-node metastasis [100].

MALAT1 is overexpressed in hepatocellular carcinoma (HCC) primary samples and cell lines [101], and its expression correlated with advanced tumor stages and reduced overall survival of HCC patients [102]; moreover, high MALAT1 levels correlated with a major risk of HCC recurrence after liver transplantation [103]. MALAT1 was found upregulated in human primary colorectal cancer (CRC) tissues with lymph-node metastasis. Clinically, high levels of MALAT1 and other Lnc-RNAs, including AFAP1-AS1, BCAR4, H19, HOXA-AS2 and PVT1, were predictive of poor prognosis of CRC patients [104]. 

Significant advances have been also achieved in developing therapeutic reagents for drugging oncogenic Lnc-RNAs in tumor cells, and novel approaches are being addressed to design and develop small molecules targeting Lnc-RNAs. Targeted genetic deletion of MALAT1 by zinc finger nucleases, as well MALAT1 therapeutic targeting by synthetic oligonucleotides, including siRNAs and the newly developed LNA gapmeR ASOs, have established the oncogenic role of this Lnc-RNA and its druggability for therapeutic purposes [105].

In this review, we show that Lnc-RNAs MEG3 and MALAT1 are involved in five and three relevant pathways of diabetic retinopathy, respectively. Therefore, both are good candidates to study. Among the 21 human investigations shown in Table 2, the human tissue analyzed was provided from blood samples, plasma, aqueous humor, epiretinal membranes and vitreous samples. These types of studies are not difficult to carry out. They can be performed in the future to find Lnc-RNA biomarkers related to retinopathy severity or associated with non-responding anti-VEGF therapy in macular edema.

To date, we have not seen a report or a registered interventional clinical trial on diabetic retinopathy targeting Lnc-RNAs in the literature. New therapies will probably become more important in the context of personalized medicine, such as adjuvant therapies targeting specific Lnc-RNAs. Further research is needed to analyze the role of Lnc-RNAs in diabetic retinopathy.

## Figures and Tables

**Table 1:** Diabetic retinopathy at the molecular level. A brief overview.

	Overproduction of Molecules	Mechanisms Involved	Site/Effect of Damage	References
Oxidative Stress	ROS AGEs	PKC, NFkB NrF2, TNF-α	mitochondria capillary cells	[8,9]
Neurodegeneration	Caspases, Bax, Bak	NFkB, SIRT1 Akt, Cox2, TGF-β	RGCs Pericytes	[4,10,11,12,13,14]
Inflammatory Process	TNF-α, IL-6, IL-8, IL-1β, iNOS, ICAM1, Complement factor	PKC—NFkB Müller cells	capillary cell death, increase vascular permeability	[5,15,16,17,18,19,20,21,22,23]
Angiogenesis	VEGF	Ischemia, PEDF	hypoxia, vitreo-retinal neovascularization	[4,5,24]

**Table 2:** Lnc-RNA effects and targets in high glucose, hyperglycemia, and diabetes. In references, (a) corresponds to animal studies, (c) to cell studies, (h) to human studies, (r) to review articles.

Lnc-RNA	Full Name	How is Lnc-RNA Expression Affected in High-Glucose/Hyperglycemia/Diabetes Conditions?	Effects and Targets	References
Lnc MALAT-1	Metastasis-Associated Adenocarcinoma Transcript 1	Upregulation in retinal endothelial cells and diabetic retinas. Upregulation in the vitreous humor, aqueous humor samples and fibrovascular membranes of diabetic patients.	Inflammatory molecules (IL-6, Tnf-α, IL-1Beta, MCP-1). Neovascularization-related proteins (VEGF, MMP2 and MMP9). Related to GRP78 producing angiogenesis and inflammation in hRVECs. Regulate cell proliferation via p38 MAPK signaling pathways. Binds Sp1, avoiding Nrf2 nuclear movement, impeding the transcription of antioxidant response enzymes (HO-1 and Sod2). Binds to miR-125b, producing VE-cadherin activation. Binds to miR-203a-3p, elevating HIF-1alpha and VEGFA. Binds to miR-200b-3p, producing proliferation, migration and tube formation of hRMECs.	[30] (a), [31] (a, c) [32] (c) [33] (h) [34] (a, c) [35] (c) [36] (a, c), [37] (c), [30] (a), [30] (a), [38] (a, c)
Lnc MEG3	Maternally expressed gene 3	Decreased in serum of diabetic patients and HG-treated RPE cells. Reduced in retinal endothelial cells and diabetic retinas.	MEG3 overexpression reduces VEFG and TGF-Beta1. MEG3 knockdown increases proliferation, migration and tube formation of retinal endothelial cells. MEG3 reduction increases retinal angiogenesis and can aggravate vascular leakage and inflammation. Methylation of CpG islands of the MEG promoter by DNMT1 leads to PI3K/Akt/mTOR signaling pathway activation, promoting endothelial–mesenchymal transition. MEG3 activates PI3K/Akt signaling pathway and regulates retinal endothelial cell events related to angiogenesis. MEG3 decreases the level of miR-233-3p, partially repressing the progression of DR and suppressing proliferation of hRECs. Lnc MEG3 expression into the vitreous cavity reduced Fox01 (oxidative stress, proliferation, apoptosis, differentiation and autophagy regulator) and IL-1Beta. Regulates miR-19b, suppressing cell apoptosis and enhancing cell viability in hRMECs. Binds to miR-34a, promoting the expression of Sirt1 and inhibiting NF-kB pathway, inflammatory response (IL-1-Beta, IL-6 and TNF-α) and apoptosis (Bcl-2/Bax ratio) in Müller cells and ARPE-19 cell line. Possibly binds to miR-204, promoting Sirt1 pathway. Targets miR-93, increasing Nrf2 in ARPE-19 cells. Consequently, it inhibits apoptosis (inhibits cleaved caspase-3 and Bcl2 and increases Bax) and inflammation (decreases IL-6 and TNF-α).	[39] (a, c, h) [40] (a), [39] (a, c), [41] (c), [42] (c), [43] (c, h), [44] (a, c), [45] (a, c), [46] (a, c, h), [47] (a, c, h), [46] (a, c, h), [48] (c), [49] (a, c), [50] (c, h), [34] (c)
Lnc MIAT	Myocardial-infarction-associated transcript Retinal non-coding RNA 2	Upregulated MIAT expression in plasma, retinal endothelial cells and Müller cells. Upregulated MIAT levels on fibrovascular membranes of diabetic patients. MIAT expression increased in hRPE cells cultured with HG.	Associated with cell proliferation, apoptosis and migration. Implicated in the regulation of vascular function, angiogenesis and vascular leakage. Upregulated MIAT produces microvascular dysfunctions (blood flow disruption, basement membrane thickening, pericyte loss and acellular capillaries). Regulator of retinal neurodegeneration. Activates TGF-Beta 1 pathway, reducing cell viability. Upregulates TGF-Beta expression in the aqueous humor in diabetic patients. Targets miR-29b, reversing Müller cell apoptosis. Binds to miR-342-3p to regulate caspase-1 expression and consequent pericyte pyroptosis. Binds to miR-150-5p, modulating VEGF expression at a transcriptional level in retinal endothelial cells. MIAT is regulated by C-myc, releasing IL-1Beta, TNF--α and IL-6 through TXNIP.	[51] (a, c), [38] (a, h), [52] (a, c), [53] (c, h), [54] (a, c), [55] (a, c), [56] (c, h) [57] (a, c)
Lnc H19	Derives from paternally imprinted *H19* gene	Downregulated in retinal epithelial cells with high glucose. Downregulated in vitreous humor samples from patients with PDR.	Overexpression of H19 inhibits inflammation in ARPE-19 cells. Binds XBP1-suppressor miR-93, increasing XBP1 which reduces inflammation (TNF--α and other inflammatory mediators) and apoptosis. Binds to miR-19b and upregulates SIRT1, reducing the expression of TNF-α, IL-1Beta and IL-6 in ARPE-19 cells. H19 overexpression blocks the MPK-ERK1/2 pathway, preventing glucose-induced endothelial–mesenchymal transition by suppressing TGF-Beta 1.	[58] (c), [48] (c), [46] (a, c, h), [59] (r), [52] (c)
Lnc HOTAIR	HOX Transcript Antisense RNA	Increased in diabetic retinas and high-glucose-stimulated RECs.	Related to proliferation, invasion, migration and permeability of HG-stimulated RECs. Related to acellular capillaries and vascular leakage in vivo. Contributes to glucose-induced mitochondrial and DNA damage. Facilitates the epigenetic activation of VEGF-A. VE-cadherin transcription inhibition.	[59] (c), [60] (a, c), [61] (a, c, h)
Lnc ANRIL	Lnc-antisense non-coding RNA in the INK4 locus	Upregulated in diabetic retinopathy and retinal tissues of DR rats.	Direct and indirect role as a recruiter of chromatin remodeling complexes, upregulating VEGF mRNA expression. Regulates NF-kB and IL-1, IL-6 and MCP-1. Related to apoptosis in retinal tissues.	[62] (a, c) [63] (a)
Lnc BANCR	B-Raf proto-oncogene, serine/threonine kinase-activated non-protein coding RNA	Plasmatic levels allow distinguishing between diabetic patients without obvious complications.	Possible biomarker for diabetic retinopathy. BANCR overexpression inhibited apoptosis in ARPE-19 cells under high glucose treatment.	[64] (c, h) [65] (c, h)
Lnc SNHG16	Small nucleolar RNA host gene 16	Upregulated after HG exposure in a dose- and time-dependent pattern.	Associated with hRMEC proliferation. Related to HIF-1alpha and VEGF expression. Binds to miR-146a-5p and miR-7-5p related to NF-kB and PI3K/AKT pathways.	[66] (c) [67] (c)
Lnc HOTTIP	HOXA transcript at the distal tip	Upregulated in retinal vascular cells and retinas of diabetic animals.	Promotes retinal inflammatory processes by activating p38-MAPK. Related to neovascularization and tube formation on vascular endothelial cells.	[68] (a, c) [68] (a, c)
Lnc NEAT1	The nuclear paraspeckle assembly transcript 1	Downregulated in Müller cells under diabetic conditions. Increased in hRECs under HG.	Related to BNDF expression, promoting cell differentiation, inhibiting inflammation and protecting photoreceptors and RGCs. Related to TGF-beta1 and VEGF signaling.	[69] (a, c), [70] (a, c, h)
Lnc BDNF-AS	Brain-derived neurotrophic factor antisense	Abundantly expressed in retina. Upregulated in RPE cells exposed to high glucose.	BDNF antisense. Related to ischemic injury in RGCs and apoptosis.	[71] (c)
Lnc HEIH	Hepatocellular Carcinoma Upregulated EZH2-Associated	Highly expressed in serum of DR patients. Increased expression on ARPE-19 cells exposed to HG.	Related to cell injury and apoptosis (releasing cytochrome C and enhancing the caspase-3 pathway). Binds to miR-939, increasing VEGF and consequently PI3K/AKT signaling pathway activation.	[72] (c, h)
Lnc IGF2-AS	Insulin-like growth factor 2 antisense transcript	Upregulated on ARPE-19 cells with HG in a concentration-dependent manner.	Related to apoptosis (caspase-9). May act through AKT signaling pathway.	[73] (c)
Lnc SNHG7	Small nucleolar RNA host gene 7	Downregulated under HG exposure.	Suppress cell proliferation, migration and angiogenesis. Inhibits EndMT. Acts through miR-543/SIRT1. Binds to miR-34a-5p/XBP1, avoiding EndMT and angiogenesis in HG-treated hRMECs and retinal inflammation and Müller glia activation in DR.	[74] (c), [75] (c), [74] (c), [76] (c)
Lnc SNHG4	Small Nucleolar RNA Host Gene 4	Downregulated in DR. Not downregulated in diabetic patients without obvious complications.	Related to protection against cytokines production, inflammation and apoptosis. Binds to miR-200b/Oxr1.	[77] (c)
Lnc RPSAP52	Ribosomal Protein SA Pseudogene 52	Lower plasma levels in diabetic patients’ RPE cells. Downregulated in RPE cells exposed to HG.	Reduces the apoptotic rate of RPE cells. Sponges miR-365 to upregulate Timp3 (decreasing apoptosis).	[78] (c, h)
Lnc KCNQ1OT1	KCNQ1 overlapping transcript 1	Higher in aqueous humor of DR patients.	Promotes cell proliferation and angiogenesis in hRECs. Binds to miR-1470, increasing EGFR and AKT pathway signaling.	[79] (c, h)
Lnc FENDRR	FOXF1 Adjacent Non-Coding Developmental Regulatory RNA	Increased in blood of DR patients. Increased in HG-exposed hRECs.	Increases the expression of FOXF1. Promotes proliferation, migration, capillary formation and VEGF expression.	[80] (c, h)
Lnc TDRG1	Human testis development-related gene 1	Overexpressed in fibrovascular membranes of patients with PDR. Highly expressed in hRECs exposed to HG.	Related to endothelial cell dysfunction and VEGF expression.	[81] (c, h)
Lnc UCA1	Urothelial-cancer-associated 1	Upregulated in endothelial cells in DM and in diabetic nephropathy in rats. Upregulated in fibrovascular membranes and in the blood of patients with DR.	Binds to miR-624-3p, leading to cell proliferation, migration and angiogenesis by promoting VEGF-C expression in endothelial cells.	[82] (c, h)
